# Lung ultrasound and coagulation activation in COVID-19 pneumonia

**DOI:** 10.1007/s40477-025-01055-2

**Published:** 2025-07-29

**Authors:** Alice Brighenti, Livia Masi, Daniela Agostinelli, Nicola Venturoli, Sofia Maria Bakken, Fabrizio Giostra, Andrea Boccatonda, Carla Serra

**Affiliations:** 1 Diagnostic and Therapeutic Interventional Ultrasound Unit, IRCCS Azienda Ospedaliero-Universitaria di Bologna, 40138 Bologna, Italy; 2 Emergency Department, IRCCS Azienda Ospedaliero Universitaria di Bologna, 40138 Bologna, Italy

**Keywords:** COVID-19, Ultrasound, LUS, Pneumonia, SARS-CoV-2

## Abstract

**Purpose:**

To evaluate the correlation between the degree of pulmonary compromise, as assessed by lung ultrasound, and the activation of the coagulation cascade in patients hospitalized with COVID-19 pneumonia.

**Methods:**

A prospective observational study was conducted on 47 adult patients with confirmed COVID-19 pneumonia. Each patient underwent a systematic 12-zone LUS exam to calculate a total Lung Ultrasound Score (LUS score). Arterial blood gas analysis was performed to calculate the arterial partial pressure of oxygen to fraction of inspired oxygen (*P*/*F*) ratio and the alveolar-arterial (A-a) oxygen gradient. Laboratory tests included serum D-dimer and C-reactive protein (CRP).

**Results:**

The LUS score correlated positively and significantly with D-dimer values (*p* = 0.019; *ρ* = 0.342), CRP (*p* < 0.001; *ρ* = 0.647), and the A-a gradient (*p* < 0.001; *ρ* = 0.640). It also displayed a significant negative correlation with the P/F ratio (*p* < 0.001; *ρ* = −0.614).

**Conclusion:**

The degree of lung injury in COVID-19 pneumonia, as evaluated by LUS, correlates directly with elevated D-dimer levels, signifying increased coagulation activation. Lung ultrasound, as a bedside and noninvasive tool, could serve as a surrogate marker not only for pulmonary involvement but also for identifying patients at higher risk of thrombotic complications.

## Introduction

COVID-19, caused by SARS-CoV-2, emerged at the end of 2019 and rapidly evolved into a global pandemic [1]. The clinical spectrum of COVID-19 ranges from asymptomatic infection to severe pneumonia and ARDS [1, 2]. Although the primary manifestation involves the respiratory system, it soon became apparent that COVID-19 is a multisystem disease with cardiovascular, renal, neurologic, and hematologic involvement [1, 2]. Among the most critical aspects of its systemic complications is the derangement of the coagulation pathway, which can lead to thrombotic events in both venous and arterial systems [1, 2]. Early studies documented markedly elevated D-dimer levels in patients with severe COVID-19, pointing toward a hypercoagulable state associated with worse outcomes, including higher rates of mortality and increased likelihood of requiring intensive care support [3].

The pathophysiology of COVID19–related coagulopathy is complex, with multiple interacting processes [4, 5]. Endothelial dysfunction plays a central role, aggravated by the proinflammatory cytokine storm characteristic of severe disease [4, 6]. The interplay between inflammation and coagulation in the setting of SARS-CoV-2 infection is widely recognized: inflammatory cytokines such as interleukin-6 (IL-6) can trigger tissue factor expression, initiating the coagulation cascade, while also suppressing the natural anticoagulant pathways [6]. This process leads to elevated fibrin degradation products, such as D-dimer, which reflect the breakdown of fibrin clots [3, 7]. In many patients, abnormally high D-dimer levels have been linked to the development of venous thromboembolism (VTE), stroke, myocardial infarction, and microthrombi in the pulmonary circulation [3, 8, 9].

Chest imaging is fundamental in diagnosing, monitoring, and prognosticating COVID-19 pneumonia. Although chest X-ray and computed tomography (CT) scans [10] have been used extensively, lung ultrasound (LUS) has emerged as an attractive bedside alternative [11]. It confers several advantages over conventional imaging modalities such as lack of ionizing radiation, bedside accessibility, sensitivity for pulmonary edema and interstitial changes and rapid learning curve [12].

Given these strengths, LUS has been used extensively to evaluate and follow COVID-19 pneumonia [11]. Several scoring systems have been developed to quantify lung involvement using ultrasound, with the lung ultrasound score (LUS score) being one of the most commonly used [13]. Typically, the lungs are divided into segments or zones (such as the 12-zone protocol employed in this study), and each zone is assigned a score based on the presence and severity of B-lines, consolidations, and other pathological findings [14].

In COVID-19, elevated D-dimer has been repeatedly associated with disease severity, need for mechanical ventilation, and mortality. This relationship underscores the importance of measuring D-dimer to identify patients who might benefit from early and/or escalated anticoagulation therapy [15]. In addition to D-dimer, other markers such as C-reactive protein (CRP) can shed light on the inflammatory status, while arterial blood gas analyses (e.g., P/F ratio and alveolar-arterial [A-a] gradient) inform the extent of respiratory compromise.

While the inflammatory and hypercoagulable facets of COVID-19 pneumonia have been well documented, there is less clarity regarding how these processes align with direct imaging measures of lung injury, such as the LUS score. If a clear relationship can be established between LUS findings and D-dimer levels, clinicians might be able to use LUS not only as a diagnostic imaging tool but also as a surrogate for assessing the extent of coagulation activation and overall disease severity. Such a correlation could have practical implications for risk stratification and therapeutic decision-making, particularly with regard to anticoagulation intensity.

## Study aims

The primary aim of this study was to evaluate the correlation between the degree of pulmonary compromise, as quantified by the LUS score using a 12-zone scanning protocol, and coagulation activation as reflected by D-dimer levels in patients hospitalized with COVID-19 pneumonia. Secondary objectives include assessing the relationship of the LUS score with other markers of disease severity, including CRP, the alveolar-arterial gradient (A-a gradient), and the P/F ratio. We hypothesize that an elevated LUS score will correlate directly with elevated D-dimer levels, as well as with heightened inflammation and gas exchange abnormalities.

## Materials and methods

### Study design and population

This was a prospective, observational study carried out at a tertiary care hospital during a surge in COVID-19 admissions. Patients were enrolled consecutively over a three-month period. Eligible participants were adults (≥ 18 years old) with confirmed COVID-19 pneumonia, diagnosed via a positive reverse transcriptase polymerase chain reaction (RT-PCR) test for SARS-CoV-2. COVID-19 pneumonia was determined based on clinical evaluation and radiologic or ultrasound findings suggestive of pulmonary involvement. Exclusion criteria included patients unwilling to participate, those with severe hemodynamic instability who could not undergo a bedside ultrasound at the time of inclusion, and patients with known chronic coagulopathies unrelated to COVID-19 (e.g., hemophilia, advanced liver disease). This study was also approved by the local Ethics Committee (551/2020/Oss/AOUBo/Comitato Etico Indipendente di Area Vasta Emilia Centro (CE-AVEC)). All procedures conformed to the ethical standards outlined in the Declaration of Helsinki. Written informed consent was obtained from the patients or their legal representatives when required.

The primary outcome was the correlation between the total LUS score and D-dimer values. Secondary outcomes were the correlations between LUS and CRP, A-a gradient and P/F ratio.

## Clinical data collection

Demographic information (age, sex, comorbidities) was recorded, along with data on clinical presentation, vital signs, and oxygen requirements. Respiratory parameters included oxygen saturation (SpO2), respiratory rate, and the fraction of inspired oxygen (FiO2) necessary to maintain oxygenation. Comorbidities of interest included hypertension, diabetes mellitus, obesity, chronic kidney disease, and cardiovascular disease, given their known association with worse COVID-19 outcomes.

## Lung ultrasound examination

A portable ultrasound machine equipped with a convex (2–5 MHz) or phased array probe was used to perform LUS at the bedside. All ultrasound exams were performed by clinicians trained in point-of-care ultrasound (POCUS), with at least one year of experience in LUS. A standardized 12-zone protocol was employed. Each hemithorax was divided into six zones (anterior, lateral, and posterior, each subdivided into upper and lower areas). In each zone, a single intercostal space was scanned to obtain the best acoustic window. The scoring in each zone was as follows:

Score 0: Presence of a normal A-line pattern or minimal isolated B-lines.

Score 1: Presence of multiple B-lines originating from the pleural line but not confluent.

Score 2: Presence of confluent B-lines or a “white lung” appearance.

Score 3: Presence of subpleural consolidations or large consolidations.

The sum of scores across all 12 zones gave a total LUS score that ranged from 0 (no lung involvement) to 36 (extensive lung involvement).

## Laboratory tests

D-dimer Assay: Blood samples were drawn at enrollment, and D-dimer levels were measured using a quantitative immunoturbidimetric assay. The results were reported in micrograms per liter (µg/L) fibrinogen equivalent units (FEU). Laboratory-specific reference ranges were applied. CRP was measured via immunoassay, reported in mg/L. CRP is a sensitive inflammatory marker whose levels rise sharply in the presence of infection or tissue injury. Arterial blood gas (ABG) analysis was performed. Arterial blood was drawn from the radial artery under sterile conditions. The following parameters were recorded: partial pressure of oxygen (PaO2): used to calculate the P/F ratio, defined as PaO2 (mmHg) divided by FiO2 (as a fraction); alveolar-arterial (A-a) Gradient: Calculated using the alveolar gas equation, which takes into account FiO2, atmospheric pressure, PaCO2, and the respiratory quotient (assumed to be 0.8 in most cases).

### Statistical analysis

Statistical computations were performed using SPSS (IBM Corp., Version XX). Descriptive statistics were expressed as means and standard deviations (SD) or medians and interquartile ranges (IQR) for continuous variables, and frequencies (%) for categorical variables. Normality was checked using the Shapiro–Wilk test. Depending on the distribution, comparisons were made using parametric (Student’s *t*-test, Pearson’s correlation) or nonparametric (Mann–Whitney *U* test, Spearman’s rank correlation) methods. Correlation Analysis: Given the nature of our data, nonparametric Spearman’s rank correlation coefficients (*ρ*) were calculated to assess associations between the LUS score and D-dimer, CRP, A-a gradient, and P/F ratio. The significance threshold was set at *p* < 0.05.

## Results

A total of 47 patients met the inclusion criteria and were enrolled during the study period (Table 1). Their mean age was 62 years (SD ± 14), and 53.2% (*n* = 25) were male. The most common comorbidities included hypertension (42.5%), diabetes mellitus (29.8%), and obesity (19.1%). Nearly all patients presented with fever and shortness of breath, whereas cough was reported in 66.0% of cases. Thirty patients (63.8%) required supplemental oxygen via nasal cannula or face mask at the time of the study, while seven (14.9%) were on noninvasive ventilation.

**Table 1:** Characteristics of enrolled patients based on their previous history, arterial blood gas analysis and blood exams

Characteristic	*N* (%)
Sex (M)	23 (48.9%)
Hypertension	20 (42.5%)
T2DM	6 (12.7%)
COPD	6 (12.7%)
Asthma	1 (2.1%)
Cancer	2 (4.2%)
CKD	5 (10.6%)
TIA/Stroke	3 (6.3%)

*T2DM* type 2 diabetes mellitus, *COPD* chronic obstructive pulmonary disease, *CKD* chronic kidney disease, *TIA* transient ischemic attack

**Table:** 

Patients’ characteristics	Mean ± SD
AGE (years)	65.3 ± 18.3
CR (BPM)	89.9 ± 17.1
RR (A/MIN)	20.2 ± 5.7
SpO_2_	95.2 ± 4.0
PaO_2_ (mmHg)	72.8 ± 19.1
PaCO_2_ (mmHg)	33.4 ± 6.7
SaO_2_	95.9 ± 5.8
P/F	332.8 ± 91.0
LAC (mmol/L)	1.2 ± 0.9
A-a O_2_ GRADIENT	37.7 ± 18.3
WBC (× 10^3^/μL)	7.0 ± 3.5
APTT	1.14 ± 0.64
INR	1.6 ± 7.2
LDH (U/L)	239.5 ± 192.0
PCT (ng/mL)	3.49 ± 17.5
CRP (mg/dL)	4.7 ± 7.1

*CR* cardiac rate, *RR* respiratory rate, *SpO₂* peripheral oxygen saturation, *PaO₂* partial pressure of oxygen in arterial blood, *PaCO₂* partial pressure of carbon dioxide in arterial blood, *SaO*_*2*_ arterial oxygen saturation, *P/F* ratio of arterial oxygen partial pressure to the fraction of inspired oxygen, *LAC* lactate, *A–a O₂ gradient* alveolar-arterial oxygen gradient, *WBC* white blood cell, *APTT* activated partial thromboplastin time INR International Normalized Ratio, *LDH* lactate dehydrogenase, *PCT *procalcitonin, *CRP* C-Reactive protein.

## Lung ultrasound findings

LUS scores ranged from 2 to 29 (median: 16; IQR: 10–22). No patient had a completely normal lung ultrasound (LUS score of 0). Subpleural consolidations and widespread B-line patterns were frequently observed in patients with higher LUS scores. The distribution of abnormal findings often involved posterior and lateral zones, reflecting a common pattern of COVID-19 pneumonia.

## Laboratory results

The median D-dimer was 1200 µg/L FEU (IQR: 800–2,000 µg/L), with levels above the laboratory upper reference limit (> 500 µg/L) in 74.5% of patients. The median CRP was 58 mg/L (IQR: 24–110 mg/L), with higher values seen in patients requiring more intensive respiratory support. The P/F ratio ranged from 110 to 450 (median: 220; IQR: 170–280). Patients with P/F ratios below 200 were considered to have moderate-to-severe gas exchange impairment. The A-a gradient values ranged from 10 to 60 mmHg (median: 38 mmHg; IQR: 28–46 mmHg), suggesting variable degrees of ventilation-perfusion mismatch.

## Correlations

**Fig. 1:**
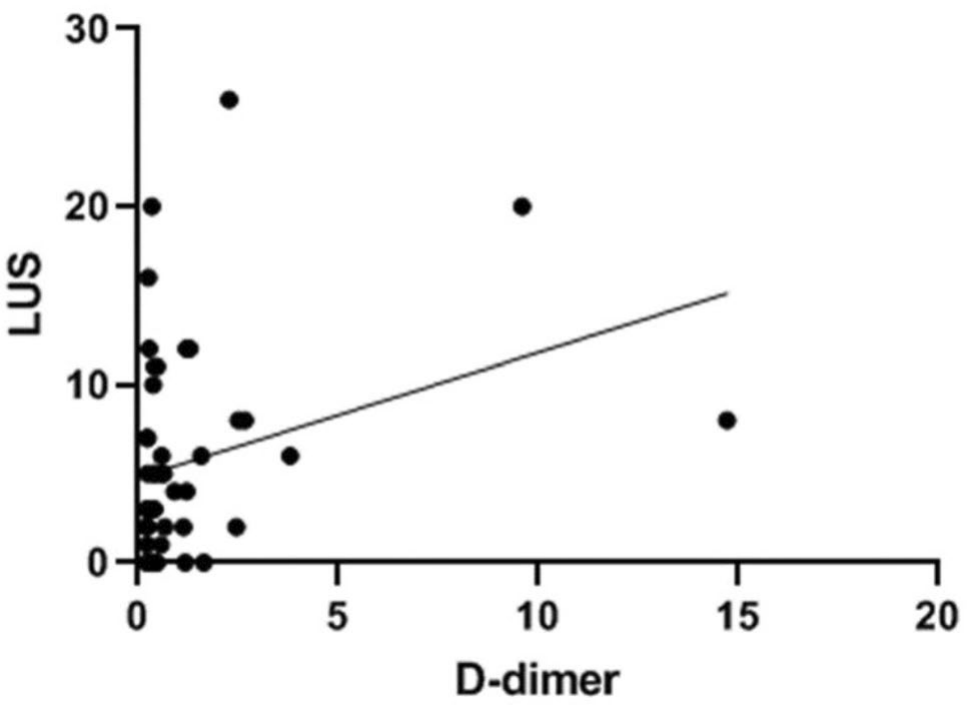
Correlation between lung ultrasound score (LUS) and D-dimer levels

**Fig. 2:**
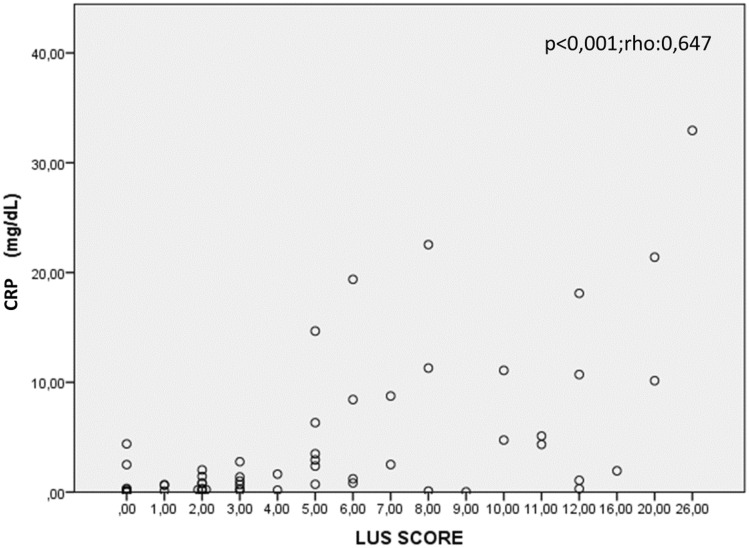
Correlation between lung ultrasound score (LUS) and C-reactive protein (CRP) levels

Spearman’s rank correlation revealed a direct and statistically significant association between LUS score and D-dimer value (*ρ* = 0.342, *p* = 0.019) (Fig. 1). Patients with higher LUS scores tended to have higher D-dimer values, indicating that more extensive ultrasound-detected lung involvement was linked to greater activation of the coagulation cascade. A strong positive correlation was noted between LUS score and CRP (*ρ* = 0.647, *p* < 0.001) (Fig. 2). The correlation was also strong and positive between LUS score and A-a gradient (*ρ* = 0.640, *p* < 0.001), consistent with the premise that increased lung ultrasound abnormalities mirror worsening gas exchange. The correlation was negative between LUS score and *P*/*F* ratio (*ρ* = −0.614, *p* < 0.001). Higher LUS scores were associated with lower *P*/*F *ratios, reinforcing that extensive ultrasound findings are reflective of impaired oxygenation. No statistically significant differences in the magnitude of these correlations were noted when stratifying by patient subgroups, such as the presence of hypertension or diabetes, although the study was not powered to detect subgroup-specific differences.

## Discussion

Our study offers a comprehensive assessment of the relationship between LUS findings and crucial laboratory and respiratory metrics in COVID-19 pneumonia. Our results demonstrated that lung involvement—as visualized via ultrasound—correlates significantly with D-dimer levels, CRP, and indices of gas exchange abnormality (A-a gradient, P/F ratio). These findings collectively highlight that, as the degree of ultrasound-detected lung injury increases, there is a corresponding rise in both inflammatory and coagulopathic markers. The documented positive correlation between LUS score and D-dimer supports the concept that COVID-19 pneumonia involves not only the alveolar and interstitial compartments but also a systemic hypercoagulable state [16]. Mechanistically, this is consistent with the cytokine storm and endothelial damage that can stimulate platelet aggregation and clotting factor activation [17]. The presence of extensive lung involvement—whether measured via CT or ultrasound—has long been associated with more severe disease, but our results extend this knowledge by showing that more severe pulmonary compromise specifically correlates with a higher probability of thrombosis or ongoing clot formation and dissolution (as indicated by higher D-dimer) [18, 19]. LUS has emerged as a critical tool for the assessment of pulmonary conditions, including pneumonia, ARDS, and pulmonary edema. Its favorable performance in COVID-19 lies in its ability to monitor disease progression, guide patient management [20]. Moreover, based on the results of our study, LUS may be a potential surrogate for coagulopathy; while D-dimer assays remain essential, in resource-limited environments or scenarios that prevent frequent lab testing, clinicians might use trends in the LUS score as a proxy for disease trajectory, indirectly raising suspicion for increased coagulopathy risk [21].

Our finding of a robust correlation between LUS scores and CRP corroborates that more extensive lung lesions coincide with heightened systemic inflammation. CRP, produced in the liver in response to interleukin-6 and other proinflammatory cytokines, has previously been recognized as a predictor of severe COVID-19 [22]. The strong association between LUS score and CRP is clinically significant, suggesting that ultrasound-based lung pathology may be a practical indicator of systemic inflammatory burden.

The negative correlation between LUS score and P/F ratio, alongside the positive correlation between LUS score and A-a gradient, underscores the reliability of lung ultrasound in reflecting functional deficits in oxygenation. As pneumonia advances and alveolar consolidation or interstitial thickening increases, gas exchange deteriorates, manifested by a rise in the A-a gradient and a decline in the P/F ratio. This matches existing literature confirming that LUS correlates well with CT findings and oxygenation parameters in both ARDS and COVID-19 pneumonia [18].

Several prior studies have indicated that ultrasound-based scores correlate with clinical severity and inflammatory markers in COVID-19 pneumonia [23–25]. Our work adds to these by specifically focusing on the link between LUS and D-dimer. Previous studies have largely emphasized imaging correlations with CRP or IL-6. In contrast, our results delineate how a relatively simple, semi-quantitative lung ultrasound scoring system can reflect a key element of COVID-19 pathophysiology—thrombotic risk.

## Limitations

Although statistically significant results were obtained, our sample comprised only 47 patients. Larger, multicenter studies could provide greater statistical power and more robust evidence. All patients were treated in a single tertiary-care hospital, which may limit the generalizability of our findings to other healthcare settings. This study offered a cross-sectional snapshot of LUS findings and laboratory values. Serial measurements over multiple timepoints would yield further insight into how changes in lung ultrasound patterns parallel shifts in coagulation parameters. Although our ultrasound operators were trained and followed a standardized protocol, there was always a potential for inter-operator variability in image acquisition and scoring.

## Conclusions

Our findings demonstrated a clear relationship between the extent of ultrasound-detected lung involvement in COVID-19 pneumonia and the magnitude of coagulation activation, as evidenced by D-dimer levels. This correlation highlights the intertwined nature of pulmonary damage and systemic coagulopathy in the pathophysiology of COVID-19. In addition to reinforcing the value of LUS as a rapid bedside imaging tool, the study supports the potential role of LUS in helping to identify patients who may be at heightened risk for thrombotic complications. Establishing a robust correlation between bedside ultrasound findings and key laboratory indicators of coagulopathy could advance personalized management in COVID-19. It may enable clinicians to promptly identify patients at higher risk of thrombotic complications and consider more aggressive anticoagulation strategies. Furthermore, the use of LUS for ongoing monitoring could reduce dependence on more resource-intensive imaging modalities and potentially allow earlier recognition of disease progression in settings where patient transportation is difficult or risky. In tandem with inflammatory markers (CRP) and gas exchange parameters (P/F ratio, A-a gradient), the LUS score can provide a comprehensive assessment of disease severity. As a low-cost, noninvasive, and readily accessible modality, LUS can be integrated into routine care pathways for COVID-19 patients, aiding in clinical decision-making and contributing to a more nuanced understanding of disease progression [26].

## Data Availability

Data are available on request from the corresponding author.
